# Comparison of Efficacy and Safety of Azilsartan and Amlodipine Combination Versus Telmisartan and Amlodipine Combination in Hypertensive Patients: A Non-inferiority Trial

**DOI:** 10.7759/cureus.35865

**Published:** 2023-03-07

**Authors:** Ashish Dash, Bikash R Meher, Biswa M Padhy, Rashmi R Mohanty, Amruta Tripathy

**Affiliations:** 1 Pharmacology, All India Institute of Medical Sciences, Bhubaneswar, Bhubaneswar, IND; 2 General Medicine, All India Institute of Medical Sciences, Bhubaneswar, Bhubaneswar, IND

**Keywords:** fixed dose combination, blood pressure, azilsartan, amlodipine, telmisartan, hypertension

## Abstract

Introduction

Hypertension (HTN) is one of the most common conditions encountered in daily practice in hospitals. Combination therapy is mostly initiated in the management of HTN when target blood pressure is not achieved with monotherapy. There are few studies comparing the antihypertensive effect of a combination of azilsartan and amlodipine with a combination of amlodipine and other angiotensin receptor blockers (ARBs), however, the results are contradictory. The objective of this study was to compare the efficacy and safety of the azilsartan and amlodipine combination versus the telmisartan and amlodipine combination in hypertensive patients.

Methods

The present study was a prospective, randomized, active-controlled, open-label, parallel-group clinical trial. Hypertensive patients were randomized into two groups of 25 patients each. Baseline evaluations of systolic blood pressure (SBP), diastolic blood pressure (DBP), and high-sensitivity troponin I (hsTnI) were done. Patients were reassessed after 12 weeks of drug therapy with azilsartan 40 mg and amlodipine 5 mg combination or telmisartan 40 mg once daily (QD) and amlodipine 5 mg combination QD.

Results

The response rate (defined as a reduction of more than 20 mm Hg in SBP or 10 mm Hg in DBP or both from baseline at 12 weeks) for HTN in the test group and control groups was found to be 88% and 96% respectively. The response rate of the azilsartan amlodipine group was found to be non-inferior to the telmisartan amlodipine group (odds ratio, OR, 0.31, p = 0.61) at the end of 12 weeks of drug therapy. At 12 weeks of follow-up, there was a significant decrease in SBP (p < 0.001), DBP (p < 0.001), and hsTnI levels (p < 0.001) in both groups from baseline values. However, differences between the test and control groups for blood pressure and hsTnI were found to be not statistically significant at 12 weeks of follow-up. The most commonly reported adverse effect in both groups was headache.

Conclusion

Azilsartan amlodipine combination had an 88% response rate, which was non-inferior to the telmisartan and amlodipine combination. Biomarkers such as hsTnI showed a significant decrease in both groups after 12 weeks of follow-up. However, there was no significant difference between the two groups.

## Introduction

Hypertension (HTN) is one of the most common conditions in hospitals and if not controlled leads to a host of complications such as myocardial infarction, stroke, renal failure, and ultimately death [1]. Though currently available anti-hypertensive drugs are very effective, despite that a large proportion of hypertensive patients do not achieve the therapeutic blood pressure (BP) goal with single-drug therapy [2]. Combination therapies exhibit greater effect and faster control of BP compared with each of the corresponding monotherapies and various guidelines including the Joint National Committee (JNC-8) recommend their use [3-5]. Among the various anti-hypertensive drug combinations, the combination of an angiotensin-converting enzyme inhibitor (ACEI) or angiotensin receptor blocker (ARB) and a long-acting dihydropyridine calcium channel blocker (CCB) is one of the most commonly used [6,7]. A study on Japanese hypertensive patients showed that combination therapy with either telmisartan/amlodipine or telmisartan/hydrochlorothiazide was more effective than monotherapy with ARB [8]. TEAMSTA Protect-1 trial (A TElmisartan and AMlodipine Study to Assess the cardiovascular PROTECTive effects) compared the changes in BP along with changes in hypertensive biomarkers like high-sensitivity troponin I (hsTnI), brain natriuretic peptide (BNP), and N-terminal (NT)-pro hormone BNP (NT-pro BNP) concentrations between telmisartan/amlodipine combination group and olmesartan/hydrochlorothiazide combination group. It was found in the study that a reduction in BP was associated with a decrease in biomarkers [9]. In a few studies relationship between conventional systolic blood pressure (SBP) and hsTnI was observed [9,10].

Among the available combination therapy, telmisartan and azilsartan are one of the most commonly prescribed ARBs for the treatment of HTN in India.

Azilsartan is the latest drug from the class of ARBs to be approved for HTN in India. There are few studies comparing the anti-hypertensive effect of azilsartan with other ARBs, however, the results are mixed [10-12]. In our literature search, we did not find any head-to-head comparison between a combination of azilsartan/amlodipine and telmisartan/amlodipine in controlling HTN, hence, we planned this study to explore the efficacy and safety of azilsartan/amlodipine combination and compare it with that of telmisartan/amlodipine combination in the hypertensive patients.

## Materials and methods

Ethical issues

The study was commenced following the approval from the Institutional Ethics Committee of All India Institute of Medical Sciences, Bhubaneswar (IEC/AIIMSBBSR/PG Thesis/2019-20/37). It was registered with the Clinical Trials Registry-India, Government of India’s official clinical trial registry (CTRI/2019/11/021961).

Study design

The study was a 12-week single-center, randomized, parallel-group, open-label, active-controlled, non-inferiority study conducted between October 2019 and December 2020 with a 1:1 allocation ratio. Written informed consent was obtained from eligible patients. They were randomized into two groups using computer-generated random codes and the allocation sequence was concealed. One group of patients received azilsartan 40 mg and amlodipine 5 mg combination and another group received telmisartan 40 mg and amlodipine 5 mg combination once daily (OD). Clinical and biochemical parameters like hsTnI were reassessed at the end of 12 weeks of treatment for both groups.

Selection of participants

Hypertensive patients of either sex between 30 and 65 years of age attending general medicine outpatient departments who did not achieve the target BP level as defined by JNC-8 with monotherapy for at least one month were included in the study. Patients diagnosed with secondary HTN, patients of hypertensive emergency and urgency, presence of hyperkalemia (potassium level ≥ 5.5 mEq), pregnant and lactating women, patients with a history of allergy to azilsartan, telmisartan, and amlodipine, clinically evident renal dysfunction (serum creatinine 1.5 mg/dL) patients with any serious hepatic, cardiovascular, or pulmonary condition, and patients diagnosed with type 1 diabetes mellitus (T1DM) or type 2 diabetes mellitus (T2DM) patients with HbA1c > 8.5% were excluded from the study.

Primary and secondary outcomes

The primary outcome measure of the study was to evaluate and compare the effectiveness (response rate) of the azilsartan-amlodipine combination against the telmisartan-amlodipine combination after 12 weeks of therapy. The secondary outcome measures were to evaluate and compare the change in systolic and diastolic blood pressures from baseline at 12 weeks in both the groups as well as to evaluate and compare the change in hsTnI level from baseline and 12 weeks. The occurrence of adverse effects was sought by the non-directive questioning of the patient at the follow-up visit. Patients had free access to the investigators for reporting any adverse effects experienced by them.

Statistical analysis

Continuous variables SBP, DBP, and hsTnI were represented as mean ± standard deviation (SD) and the categorical variable (response rate) was represented as a percentage. Comparison of mean between the groups was performed using Independent t-test/Mann Whitney U test and within the group by repeated measures ANOVA/two-sided paired t-test/Wilcoxon signed-rank test. Fisher’s exact test was used for comparing categorical variables between the groups. A ‘p’ value of <0.05 was considered statistically significant. Data analysis was carried out using Statistical Product and Service Solutions (SPSS) (IBM SPSS Statistics for Windows, Version 23.0, Armonk, NY).

Sample size calculation

Based on the previous studies, the response rate of the telmisartan and amlodipine combination was taken as 95%. The non-inferiority margin was taken as 15%. A sample size of 25 in each group was found to prove the azilsartan and amlodipine combination against the telmisartan and amlodipine combination for the above-mentioned margin assuming α error of 5% and β error of 20%.

## Results

**Figure 1:**
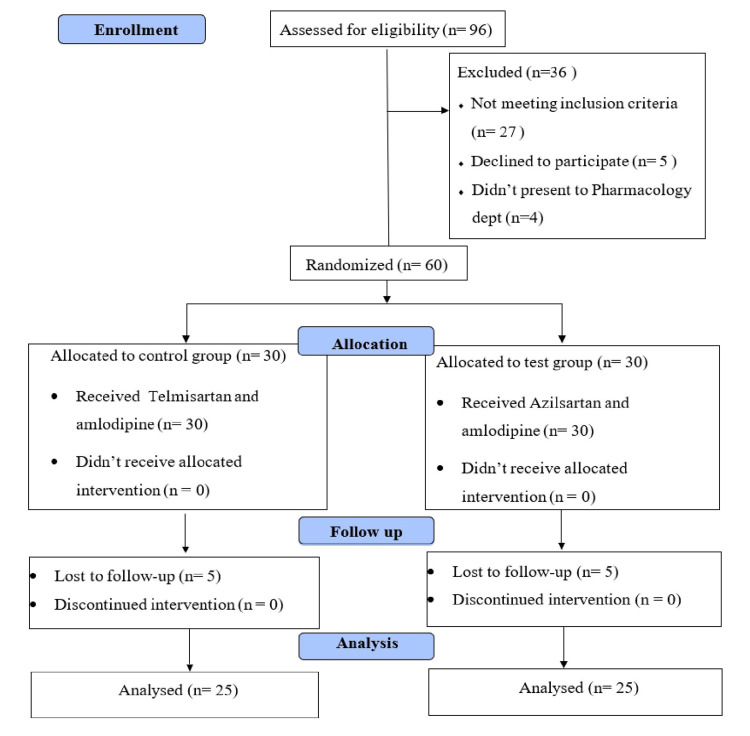
CONSORT diagram showing the flow of participants through each stage of the randomized trial (CONSORT: Consolidated Standards of Reporting Trial)

Ninety-six patients who were diagnosed with HTN were screened for the study, out of which 36 patients were excluded as they did not satisfy the inclusion criteria (Figure 1).

Patient demographic and baseline characteristics

Table 1 shows the baseline characteristics of the study participants. At baseline, there were no significant differences in age, gender, biochemical, and clinical parameters between the two groups.

**Table 1:** Baseline characteristics and clinical parameters of the patients in the two groups (^ Data presented in Median (Inter Quartile Range)) SBP: systolic blood pressure; DBP: diastolic blood pressure; BUN: blood urea nitrogen; hs cTnI: high sensitivity cardiac Troponin I

Parameter	Test group (n = 25)	Control group (n = 25)	P-value
Male	9	15	0.078
Female	16	10	0.089
Age (years)	53.68 + 8.86	52 + 9.85	0.55
Duration of hypertension in months (Median (IQR))	24 (9-36)	12 (4.8-30)	0.46
Diabetes	6 (20)	6 (24)	1.00
Hypothyroidism	2 (12)	4 (16)	0.67
Pulse rate (beats/min)	85.48 + 11.49	89.04 + 13.08	0.31
SBP (mm of Hg)	165.12 + 8.45	164.16 + 6.24	0.65
DBP (mm of Hg)	98.48 + 6.72	97.36 + 5.25	0.58
Serum Urea (mg/dL)	29.32 + 8.02	30.94 + 7.86	0.47
Serum Creatinine (mg/dL)	1.05 + 0.24	1.06 + 0.18	0.89
Serum BUN (mg/dL)	13.78 + 3.77	14.54 + 3.69	0.47
hs cTnI^ (pg/mL)	4.98 (2.34-13.82)	8.22 (1.90-15.77)	0.60

Response rate

Responders were defined as those who have a reduction of more than 20 mm of Hg in SBP at 12 weeks from baseline or a change of more than 10 mm of Hg in DBP at 12 weeks from the baseline. The response rate of the test (azilsartan and amlodipine) group was found to be 88% and that of the control (telmisartan and amlodipine) group was 96%. Thus, considering a non-inferior margin of 15%, the response rate of the azilsartan and amlodipine combination group is found to be non-inferior to the telmisartan and amlodipine combination group (95% CI: 0.03 to 3.16, p = 0.61) (Table 2).

**Table 2:** Responders in two groups

Group * Responders					
		Response		Total	P value^@^
		Responders	Non-responders		
Group	Test group (Azilsartan + Amlodipine)	22	3	25	0.61
	Control group (Telmisartan + Amlodipine)	24	1	25	
Total		46	4	50	

Change in SBP

The mean SBP differed statistically significantly from baseline to 12 weeks in both the test and control groups. However, at 12 weeks of follow-up, there was no statistically significant difference in change in SBP between the test and control group (-3.04 (95% CI, -6.93 to 0.85) mm of Hg, p = 0.12) (Table 3).

Change in DBP

The mean DBP differed statistically significantly in the test as well as the control group from baseline to 12 weeks, however, at 12 weeks of follow-up, there was no statistically significant difference in change in DBP between the test and control (0.88 (95% CI, -2.12 to 3.88) mm of Hg, p = 0.56) (Table 3).

**Table 3:** Change in systolic blood pressure (SBP) and diastolic blood pressure (DBP) after 12 weeks of drug therapy

Variable	Azilsartan + Amlodipine (n=25)				Telmisartan + Amlodipine (n=25)				Between two groups at 12 weeks	
	Baseline	Follow-up	Mean difference	P-value	Baseline	Follow-up	Mean difference	P-value	Mean difference	P-value
SBP (mm of Hg)	165.12 + 8.45	142.72 + 8.73	22.40 (18.16 to 26.63)	<0.05	164.16 + 6.24	138.72 + 7.12	25.44 (21.84 to 29.03)	<0.05	-3.04 (-6.93 to 0.85)	0.12
DBP (mm of Hg)	98.16 + 6.16	86.88 + 6.00	11.28 (8.12 to 14.44)	<0.05	97.28 + 5.06	86.88 + 6.25	10.40 (7.51 to 13.29)	<0.05	0.88 (-2.12 to 3.88)	0.56

Change in hsTnI

In the amlodipine-azilsartan group, there was a significant decrease in serum hsTnI values from baseline to 12 weeks of follow-up (p < 0.001). In the amlodipine-telmisartan group also there was a significant decrease in serum hsTnI values from baseline to 12 weeks of follow-up (p < 0.001). But, the test group had no significant difference in change in serum hsTnI concentrations at 12 weeks than the control group (p = 0.27) (Table 4).

**Table 4:** Change in high-sensitivity troponin I (hsTnI) after 12 weeks of drugs therapy (Data in Median (Inter Quartile Range))

Variable	Test group (n=25)			Control group (n=25)				Difference between the two groups at 12 weeks
	Baseline	Follow-up	P-value	Baseline	Follow-up	P-value	P-value	
Serum hsTnI (pg/mL)	4.98 (2.34 to 13.82)	3.26 (1.76 to 11.26)	< 0.05	8.22 (1.90 to 15.77)	5.76 (1.22 to 12.97)	< 0.05	0.27	

Safety assessment

Safety assessment was carried out at each visit. In the test group, headache was the most common adverse effect (12%). In the control group, dizziness and headache were the most common (8% each). Causality was assessed using the WHO-UMC assessment system and all were marked as probable. None of the adverse events were serious in nature.

## Discussion

In this study, the response rate showed by the azilsartan/amlodipine combination group was non-inferior to the telmisartan/amlodipine combination group. SBP and DBP decreased significantly from baseline to 12 weeks in both groups. However, there was no significant difference between the two groups at any of the follow-ups. A study conducted by Weber et al. demonstrated that 24 hours of ambulatory BP reduction was more in the azilsartan-amlodipine combination compared to the amlodipine placebo combination after six weeks of therapy [13]. Cushman et al. found that azilsartan medoxomil plus chlorthalidone reduces BP more effectively than olmesartan plus hydrochlorothiazide in stage 2 systolic HTN [14]. However, a meta-analysis concluded that the combination of ARB and CCB had no statistically significant benefit in terms of reducing the risk of end-stage renal disease (ESRD) or cardiovascular mortality compared to ARB monotherapy [15]. In terms of changes in SBP and DBP, combination therapy resulted in a significant decrease in SBP, but no significant difference in DBP when compared to monotherapy [16].

Myocardial damage caused by elevated BP is one of the most well-studied consequences of HTN and is a significant predictor of cardiovascular outcomes in and of itself. Cardiac troponins (cTn) are released by myocardial cells in the presence of overt myocardial injury and are easily quantifiable [17]. One study found that there was a significant relation between hs-cTnI and SBP [18]. The TEAMSTA trial discovered that lowering BP in patients resulted in a decrease in hs-cTnI, BNP, and NT-pro BNP concentrations after a half-year of anti-hypertensive therapy, with ARB-CCB combination being superior to ARB-diuretic combination [9]. In our study, serum hsTnI was significantly lowered at three months from baseline in both groups, whereas, there was no significant difference in change in hsTnI levels between the groups.

Safety analysis of our study showed that headache was the most frequently reported adverse effect in the azilsartan and amlodipine group (12%). Dizziness and headache were the most frequently reported side effects in the telmisartan and amlodipine groups (8% each). In a study involving azilsartan medoxomil 40 mg and amlodipine 5 mg vs placebo and amlodipine 5 mg, adverse events occurred at an almost similar rate in the placebo and amlodipine 5 mg combination group (47%) and azilsartan medoxomil 40 mg and amlodipine 5 mg combination group (48%) groups [13].

This study has some inherent limitations. It was an open-label study; so, the chances of bias may be more. It was carried out in a small population and for a relatively short follow-up period for a chronic disease like HTN.

## Conclusions

This was the first head-to-head comparison between azilsartan and amlodipine combination and telmisartan and amlodipine in hypertensive patients. The response rate was found to be non-inferior between both groups. There was a statistically significant difference in change in SBP at four, eight, and 12 weeks within each group. There was a significant reduction in serum hsTnI from baseline to 12 weeks in both treatment arms. However, there was no statistically significant change between both groups at 12 weeks. Headache was the most common adverse effect across both groups. A causality assessment was done using the WHO-UMC scale and all the adverse events were found to be probable in nature. None of them were found to be serious.
